# Treatment With Melatonin After Corneal Graft Attenuates Rejection

**DOI:** 10.3389/fphar.2021.778892

**Published:** 2021-10-19

**Authors:** Ziqian Zhu, Ruiping Peng, Hongyi Shen, Lei Zhong, Siqi Song, Tao Wang, Shiqi Ling

**Affiliations:** Department of Ophthalmology, The Third Affiliated Hospital of Sun Yat-Sen University, Guangzhou, China

**Keywords:** corneal transplant, NLRP3 inflammasome, macrophages, CD4^+^ T cells, melatonin

## Abstract

**Background:** Immunologic graft rejection is the main complication of corneal transplants. This study aimed to investigate the effect of melatonin (MT) on the rejection of corneal transplantation.

**Methods:** Corneal allografts were performed by grafting corneas from BALB/C mice to C57BL/6 hosts. MT (50 mg/kg) was intraperitoneally injected into the hosts every day from the day of transplantation. The survival of grafts was observed by slit lamp biomicroscopy, and inflammatory cell infiltration was detected by hematoxylin and eosin staining and immunohistochemistry. The balance of Teff and Treg immune cells in draining lymph nodes (DLNs) was detected by flow cytometry. The levels of cytokines related to the grafts and DLNs were detected using real-time fluorescence quantitative PCR. Additionally, we used the mouse macrophage line RAW264.7 to study the effect of MT on the activation of NLRP3 inflammatory body.

**Results:** MT treatment improved the graft survival rate, reduced inflammatory cell infiltration in the graft, decreased the percentage of Th1/Th17 cells in the DLNs, and increased the percentage of Treg cells. Melatonin inhibited the activation of the NLRP3 inflammasome, thereby reducing the expression of IL-1β and other related proinflammatory cytokines such as MCP-1, MIP-1, NLRP3, ASC, TNF-a and VEGF-A (all *p* < 0.05).

**Conclusion:** Our study demonstrates that MT promotes the survival of mouse corneal grafts by inhibiting NLRP3-mediated immune regulation, reducing immune cell activation and cell migration, and inhibiting the production of inflammatory-related cytokines. Treatment with MT might provide a potential clinical therapeutic target for corneal transplantation.

## Introduction

Currently, more than 10 million people worldwide suffer from corneal blindness (Amouzegar et al., 2016). Corneal trauma and disease can cause irreversible damage to the normal structure and physiological function of the cornea, and patients usually require corneal transplantation. Corneal allograft rejection is a major complication leading to decreased vision after transplantation. The incidence of postoperative immune rejection of inflammation and vascularized cornea can reach as high as 50% (Armitage et al., 2019). Although the application of corticosteroids and other immunosuppressive agents has improved the survival rate after corneal transplantation, the long-term use of these drugs can lead to serious complications such as glaucoma, cataract and weight gain (Nguyen et al., 2007). Therefore, it is necessary to determine new therapeutic targets according to the mechanism of corneal transplantation rejection.

Allograft failure is mainly caused by immune-mediated graft destruction, a complex and highly coordinated process involving interactions between innate and adaptive immune cells (Ingulli, 2010). The migration of immune cells to the graft site and local lymphoid tissue plays a considerable role in the pathogenesis of post-transplant rejection (Murooka and Mempel, 2012). The innate immune cells involved in the immune rejection reaction in the early stage can induce the subsequent activation of adaptive immune cells. Macrophages are an indispensable antigen presenting cell (APC). After activation, these cells can regulate the clearance of apoptotic neutrophils and promote the activation and proliferation of T cells (Yamada et al., 2005). Activated T cells, especially CD4+T cells, which are important adaptive immune cell, can enhance the migration of innate immune cells (macrophages and neutrophils, etc.), promote the explosion of inflammatory cytokines, and trigger transplant rejection. This reaction indicates that once the adaptive immune response is activated, it can amplify the influx of APCs, triggering a strong hypersensitivity reaction and exacerbating rejection (Slegers et al., 2004).

The NOD-like receptor protein (NLRP3) inflammasome is a protein complex composed of NLRP3, apoptosis-related speckle-like protein (ASC) and caspase-1. It promotes interleukin (IL-1β) secretion and release of IL-18, which in turn triggers an inflammatory response and induces pyroptotic cell death (Weigt et al., 2017). Cytokines derived from macrophages and treated with caspase 1 can activate the adaptive immune response in mice. The activation of these cytokines that cause a series of triggering responses requires NLRP3 (also confirmed in serum samples of patients with pancreatitis) (Sendler et al., 2019). NLRP3 promotes CD4+T cell activation and macrophage differentiation during transplant rejection, induces inflammatory chemokine secretion, and exacerbates immune-mediated rejection injury (Wei et al.; Tian et al., 2021). Therefore, NLRP3, as an important regulator of adaptive immunity, may be a potential therapeutic target.

Melatonin (MT), also known as N-acetyl-1-5-methoxychromatamine, is a neural hormone that mainly acts on circadian rhythms; however, it also has immunomodulatory, antioxidant and anti-inflammatory properties (Fildes et al., 2010; Esteban-Zubero et al., 2016). Many *in vitro* and *in vivo* studies have shown that MT inhibits NLRP3 inflammasome activity *via* various intracellular signaling pathways (Ehirli et al., 2021). Melatonin exerts a protective effect against acute lung injury by negatively regulating the NLRP3 inflammasome in macrophages (Ma et al., 2018). Furthermore, MT suppresses T cell cytokine production and NLRP3 inflammasome activation and ameliorates airway inflammation (Wu et al., 2019). However, it is not clear whether MT plays a role in regulating the innate and adaptive immune responses during corneal graft rejection.

Our study aimed to further demonstrate that MT inhibits NLRP3 in a corneal transplantation model, thereby affecting the migration of both innate and adaptive immune cells to the corneal and cervical lymph nodes, as well as the secretion of inflammatory factors and chemokines, thereby modulating the immune response to promote rejection after transplantation.

## Materials and Methods

### Animals

Female C57BL/6 and BALB/c mice (6–8 weeks of age) were used in this study. The animals were placed in a pathogen-free sterile animal room. All experiments were carried out by closely observing the ARVO Statement for the Use of Animals in Ophthalmic and Vision Research, and the approved protocols by the Institutional Animal Care and Use Committee of Zhongshan Ophthalmic Center at Sun Yat-sen University (Ethics approval number: 2021–057).

### Mouse Model of Corneal Transplantation and Pharmaceutical Treatments

According to the standard protocol, penetrating allograft corneal transplantation was performed between BALB/C (donor) and C57BL/6 (recipient) mice that were fully mismatched (Tomoyuki et al., 2019). For all animals, 1% pentobarbital sodium (40–50 mg/kg) was intraperitoneally administered as general anesthesia. Briefly, the bilateral central corneas (2 mm diameter) of BALB/C mice were excised and fixed in the central graft beds of the left eyes of C57BL/6 mice, which were prepared using a 1.5 mm trephine (Suzhou Mingren Medical Equipment Co., Ltd., China) with eight interrupted sutures (11–0 nylon, Alcon, United States). Air was used to restore the anterior chamber of the eye, and antibiotic ointment was applied locally after the operation. Recipient mice were randomly assigned to experimental groups, and intraperitoneally administered with 50 mg/kg/day MT (Selleckchem, Houston, TX, United States); 0.15 ml injections were given at 09:00 h (Del Sole et al., 2011) once a day for 9 days. Meanwhile, other recipient mice were administered with physiologic sterile saline (7.5 ml/kg, IP) as a vehicle control.

### Clinical Evaluation

Corneal grafts were examined with a slit-lamp microscope every day (*n* = 8 per group). According to the previously mentioned corneal transplant rejection evaluation criteria, the main analysis aimed to evaluate the degree of corneal opacity, edema, and neovascularization (Reuer et al., 2018). When the opacity score is ≥ 3 and the total score of the initial transparent graft is ≥ 5, it is defined as transplant rejection. Cases of postoperative transplant failure due to technical reasons, such as anterior chamber bleeding or endophthalmitis, were excluded. To improve the accuracy of assessing corneal graft rejection, each animal was observed and evaluated by two experienced inspectors.

### Quantitative Real-Time PCR

After grinding the corneas and DLNs tissue (Including submandibular lymph nodes, surperficial cervical lymph nodes, and internal jugular lymph nodes) on an ice box, we extracted total RNA from the samples using TRIzol reagent (Invitrogen, Waltham, Massachusetts, United States), and measured the concentration using a spectrophotometer (ND-1000, Nanodrop Technologies, Wilmington, Texas, United States). Next, the RNA was reverse-transcribed into cDNA using Reverse transcription reaction with PrimeScript™ RT Master Mix (Perfect Real Time, TaKaRa Bio. Co., Japan), the Kit reverse transcription kit was used to synthesize the first strand of cDNA. Subsequently, we used SYBR Premix Ex TaqII (TaKaRa Bio. Co.) dye method fluorescent quantification reagent to quantify mRNA levels according to the manufacturer’s instructions. Finally, the results were analyzed by the 2-ΔΔCt method, and the expression of each gene was normalized based on the expression of GAPDH. The primers were purchased from Invitrogen (Carlsbad, California, United States), and the relevant sequences are shown in Table 1.

**TABLE 1:** Primer sequences of mouse genes for real-time RT-PCR.

MCP-1	
forward primer	TTA​AAA​ACC​TGG​ATC​GGA​ACC​AA
reverse primer	GCA​TTA​GCT​TCA​GAT​TTA​CGG​GT
MIP-1	
forward primer	TTC​TCT​GTA​CCA​TGA​CAC​TCT​GC
reverse primer	CGT​GGA​ATC​TTC​CGG​CTG​TAG
NLRP3	
forward primer	ATC​AAC​AGG​CGA​GAC​CTC​TG
reverse primer	GTC​CTC​CTG​GCA​TAC​CAT​AGA
ASC	
forward primer	GAC​AGT​GCA​ACT​GCG​AGA​AG
reverse primer	CGA​CTC​CAG​ATA​GTA​GCT​GAC​AA
IL-1β	
forward primer	TTC​AGG​CAG​GCA​GTA​TCA​CTC
reverse primer	GAA​GGT​CCA​CGG​GAA​AGA​CAC
TNF-α	
forward primer	CAG​GCG​GTG​CCT​ATG​TCT​C
reverse primer	CGA​TCA​CCC​CGA​AGT​TCA​GTA​G
VEGF-A	
forward primer	GCA​CAT​AGA​GAG​AAT​GAG​CTT​CC
reverse primer	CTC​CGC​TCT​GAA​CAA​GGC​T
IFN-γ	
forward primer	AGT​TCT​GGG​CTT​CTC​CTC​CT
reverse primer	GGC​TTT​CAA​TGA​CTG​TGC​CG
IL-17	
forward primer	CCC​TCA​GAC​TAC​CTC​AAC​CG
reverse primer	CAT​GTG​GTG​GTC​CAG​CTT​TC
TNF-α	
forward primer	CTT​GTT​GCC​TCC​TCT​TTT​GCT​TA
Reverse primer	CTT​TAT​TTC​TCT​CAA​TGA​CCC​GTA​G
FoxP3	
forward primer	CTC​TAG​CAG​TCC​ACT​TCA​CCA​A
reverse primer	CAC​CCA​CCC​TCA​ATA​CCT​CTC​T
GAPDH	
forward primer	TGA​CCT​CAA​CTA​CAT​GGT​CTA​CA
reverse primer	CTT​CCC​ATT​CTC​GGC​CTT​G

Melatonin attenuates corneal graft rejection.

### Assessment of Lymphangiogenesis and Angiogenesis in Flat-Mounted Corneas

We quantified the formation of corneal neovessels with corneal whole mounts. To produce these mounts, mice were sacrificed at a fixed time. The eyeballs were removed, the corneas were blocked with 3% bovine serum albumin in phosphate-buffered saline with 0.3% Triton X-100 for 1 h at room temperature, and then incubated overnight at 4°C with rabbit anti-mouse LYVE-1 monoclonal antibody (1:100; Abcam) or rabbit anti-CD31 (1:50; Abcam) polyclonal antibody. Next, the samples were incubated with Alexa Fluor 488-coupled donkey anti-rabbit antibody (1:800; Abcam) or anti-rabbit IgG Alexa Fluor 555 (cell signaling technology, 1:1,000) for 2 h at room temperature. The cornea was laid flat under a microscope (DMI3000 B; Leica, Wetzlar, Germany). Photos were automatically taken to reconstruct the entire image of the cornea. ImageJ software was then used (NIH, Bethesda, Maryland, United States) to outline the innermost lymph nodes of the limbus to calculate the total area of the cornea, and the area of the newborn lymphatic vessels in each mouse were subsequently calculated (Ren et al., 2020).

### Corneal Immunostainings

For the corneal inflammatory cell recruitment test, mouse eyes were embedded in a compound at the optimal cutting temperature, sequentially sectioned (8 µm) and stored at −80°C. Subsequently, the frozen sections were blocked with 3% bovine serum albumin at 37°C for 1 h. Then at 4°C, the frozen sections were inducible rat anti-F4/80 (Abcam, ab66440, 1:100) and rat anti-CD11b (Abcam, ab8878, 1:100) overnight. Sections were then washed with phosphate buffered saline and incubated with anti-rat IgG Alexa Fluor 555 (cell signaling technology, 1:1,000) and anti-rat IgG Alexa Fluor 488 (Cell signaling Technology, Germany, 1:1,000) for 1 h at room temperature, followed by DAPI (Abcam, Ab228549) solution staining. Photographs were taken with a fluorescence microscope (DM 4000B). The number of positive cells was quantified using Adobe Photoshop CC (Adobe Systems, Inc., San Jose, CA, United States) with the method described by et al. (Dai et al., 2018). All histological assessments were performed as blinded studies by the same two observers.

### Histology

On the 9th day after corneal transplantation, eyeballs were removed, embedded in paraffin and cut into 5 μm thick sections which were then stained with hematoxylin eosin.

### Flow Cytometry

For Treg/Teff cell staining (Yang, et al., 2018), live/dead cells were first labeled with antibodies to distinguish living cells (Waltham, Ma, United States). The antibodies used included; anti mouse CD4 percp-cy5.5, anti-mouse cd45-bv510, anti-mouse IL-17A APC and anti-mouse IFN-γ PE, anti-mouse TNF-α Bv421, anti-mouse Foxp3 FITC (all from BioLegend). For Teff cells, cytokine staining analysis mainly containing Th17 and Th1, the cells mixed with acetate (50 ng/ml; Sigma-Aldrich), ionomycin (500 ng/ml; Sigma-Aldrich) and brefidine (1 µg/ml; Sigma-Aldrich) were cultured together and placed in 96 well plates with cytokine secretion blockers for 4 h. Stained cells were measured using BD LSR Fortessa flow cytometer (BD Biosciences) and data were obtained and were analyzed with Flowjo 10.0 (Flowjo company, United States).

### To Use CCK-8 Kit to Detect Cell Viability

RAW264.7 mouse macrophages were purchased from the Cell Bank of the Chinese Academy of Sciences (Shanghai, China) and cultured in Dulbacco modified Eagle medium (DMEM). Cells were stored in a 0.2 ml suspension of 2 × 10^5^ cells/well in a 96-well plate, supplemented with 10% FBS and 1% penicillin-streptomycin and treated with melatonin (0.8, 4, 20, 100, 500, and 2500 µM) in a CO2 incubator at 37°C (Kos et al., 2006). After 48 h, 10 µl CCK-8 reagent (Japanese Dojindo) was added to each well and cells were further cultured at 37°C for 1–2 h. The absorbance at 450 nm in each well was measured with a microplate reader (Swedish Famasia). The experiment was repeated three times for each group.

### Enzyme-Linked Immunosorbent Assay

To measure the effect of melatonin on macrophages *in vitro*, RAW264.7 cells were stimulated with 100 ng/ml LPS for 4 h and then incubated with different concentrations of melatonin (0.8, 4, 20, 100, 500, and 2,500 µM). In addition, cell supernatants were collected at specific time points and stored at −80°C. The ELISA kit (Invitrogen) was used to measure the concentration of inflammation-related cytokines. All samples were analyzed in triplicate and measured at 450 nm wavelength.

### Statistical Analysis

The data in this study were expressed as mean ± SEM using GraphPad Prism software (GraphPad Software, La Jolla, CA, United States). The survival probability was estimated using Kaplan-Meier survival curve and evaluated using logarithmic rank test. Student’s t-test, Mann–Whitney test, and one-way analysis of variance (ANOVA) were used to compare differences between groups. Statistical significance was set as *p* < 0.05.

## Results

### MT Treatment Prolonged Corneal Allograft Survival

**FIGURE 1:**
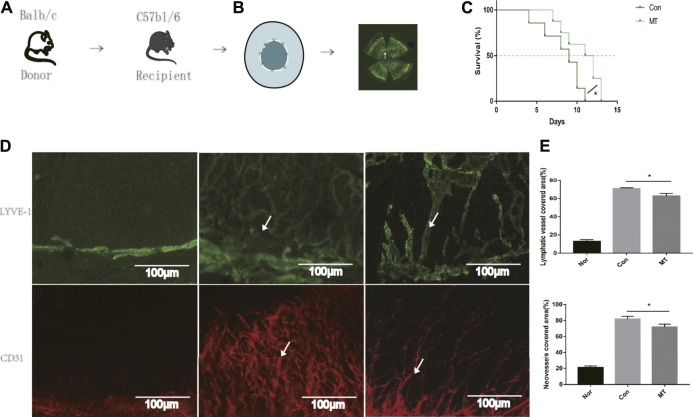
MT therapy alleviates corneal graft rejection and corneal lymphangiogenesis and angiogenesis. **(A)** Establishment of the corneal transplantation model. **(B)** Combined image of all lymphatic vessels reaching the interface 9 days after corneal transplantation (white arrows). **(C)** Postoperative corneal graft survival curve of mice (CT; MT:50 mg/kg). MT injection delayed corneal graft rejection compared to the saline group (*n* = 8 per group). **(D)** On day 9 of transplantation, LYVE-1 and CD31^+^ staining (white arrows) were performed on the entire cornea of mice. In the normal mouse eye, lymphatics and neovessles are located only at the margin of the cornea and the corneal lymphangiogenesis and angiogenesis developed after transplantation (Scale: 100 µm). **(E)** MT inhibited corneal lymphangiogenesis and angiogenesis (*n* = 3 per group) (Nor: Normal group; Con: control group; MT: treated with MT). *p < 0.05, **p < 0.01, ***p < 0.001.

Two of the eight allografts remained non-rejected in the MT group, whereas none of the eight allografts survived in mice treated with physiological saline. The average time of transplant rejection in the control group was 8.28 ± 0.94 days, while that in the MT-treated group was 10.63 ± 0.82 days. Compared with the control group, intraperitoneal injection of MT delayed immune rejection (*p* < 0.05, Figures 1A–C, *n* = 8 per group).

Given that lymphangiogenesis and angiogenesis are critical factors aggravating graft rejection (Hos et al., 2014), in our experiments, we used LYVE-1 (a specific antibody used to label lymphatic endothelial cells) and CD31 (a vascular endothelial cell marker) for immunofluorescence staining of corneal grafts. Lymphatic and Blood vessels have been shown to be located only at the limbus of the cornea in normal mice (Maruyama et al., 2005). On the 9th day after transplantation, immunofluorescence of the whole cornea revealed new lymphatic and blood vessels were present in the cornea beds. Compared with the saline group, the area of corneal lymphatic and corneal neovessels in the MT treated group was reduced (*p* < 0.05, Figures 1D,E). The results showed that MT inhibited lymphangiogenesis and angiogenesis after corneal transplantation.

### MT Application Inhibited Inflammatory Cells, F4/80+, and CD11b + Positive Cells in Corneas

**FIGURE 2:**
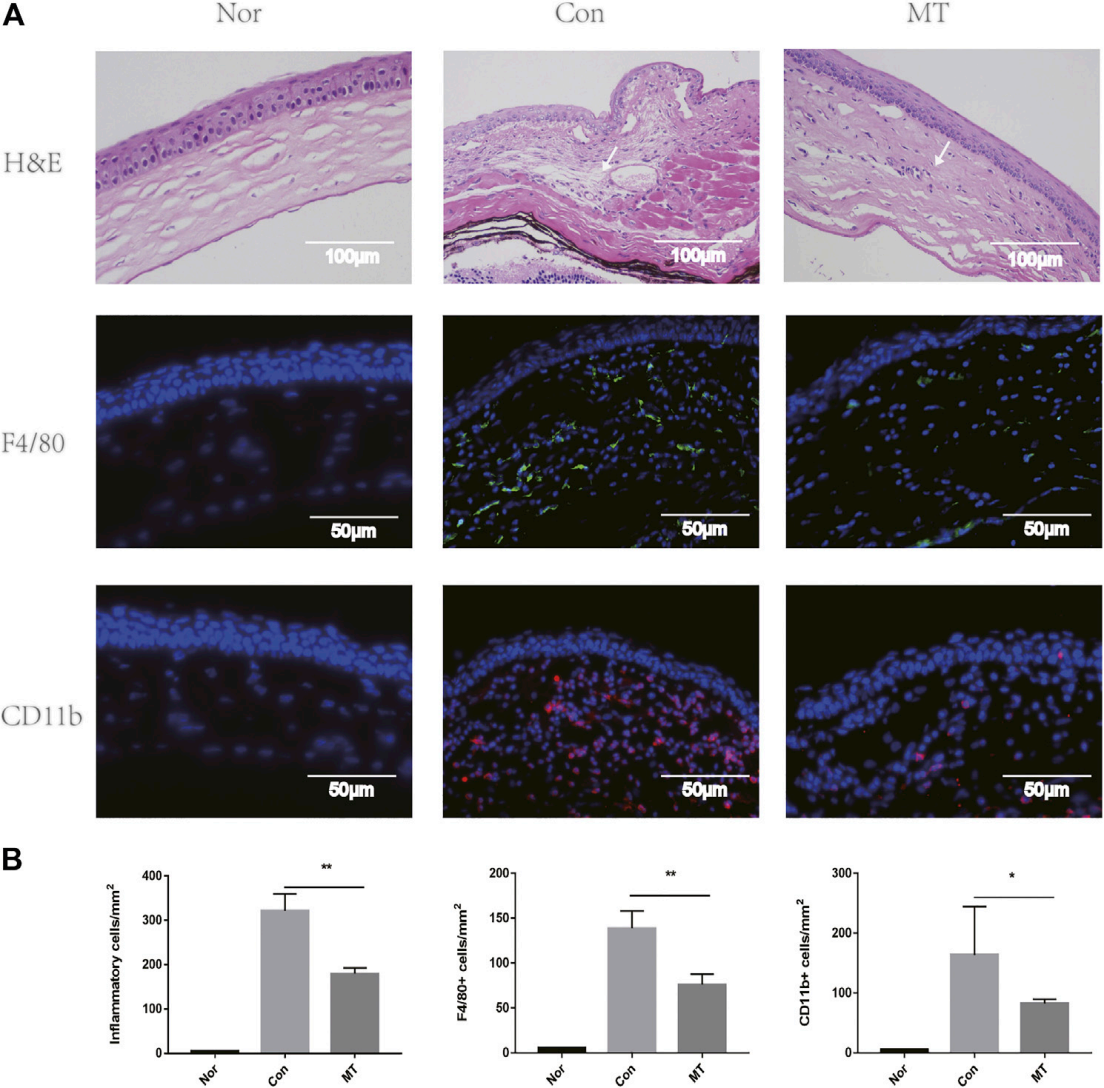
MT inhibits inflammatory cells, F4/80, and CD11b recruitment. **(A)** At day 9 of transplantation, histopathology revealed less inflammatory cell (white arrows) infiltration in corneal grafts in the MT group (representative images for each group). Fluorescence images showing infiltration of F4/80 and CD11b in the grafts of each group at day 9. Corneal sections were stained with immunofluorescent antibodies for positive cells, as shown (Scale: 50 µm). **(B)** Count of positive cells in corneal grafts of each group on day 9 (*n* = 3 per group). (Nor: Normal group; Con: control group; MT: treated with MT). *p < 0.05, **p < 0.01, ***p < 0.001.

The corneal grafts were examined and scored using a slit lamp biometric microscope (Zhong et al., 2018). On the 9th day after transplantation, mouse corneal grafts treated with saline showed turbidity and edema. HE staining showed substantial infiltration of immune cells, collagenous fiber disorder, and stromal edema in the saline group, in which grafts were rejected. In contrast, grafts that were not rejected in the MT group showed mild turbidity, few vessels, and edema, while HE staining showed a significant reduction in inflammatory cell infiltration. It has previously been demonstrated that macrophages play an important role in inducing lymphangiogenesis and angiogenesis in a mouse transplantation model (Ji and Sciences, 2012). To further understand the effect of MT on lymphangiogenesis and angiogenesis, we used F4/80, CD11b immunofluorescence staining in the cornea to compare the groups. The number of CD11b [which controls monocyte migration (Zheng et al., 2015)] and F4/80 positive cells increased in corneal graft rejection, and MT treatment attenuated inflammatory cells (Figure 2).

### MT Inhibits the Expression of Chemokines and Proinflammatory Factors During Allokeratoplasty

**FIGURE 3:**
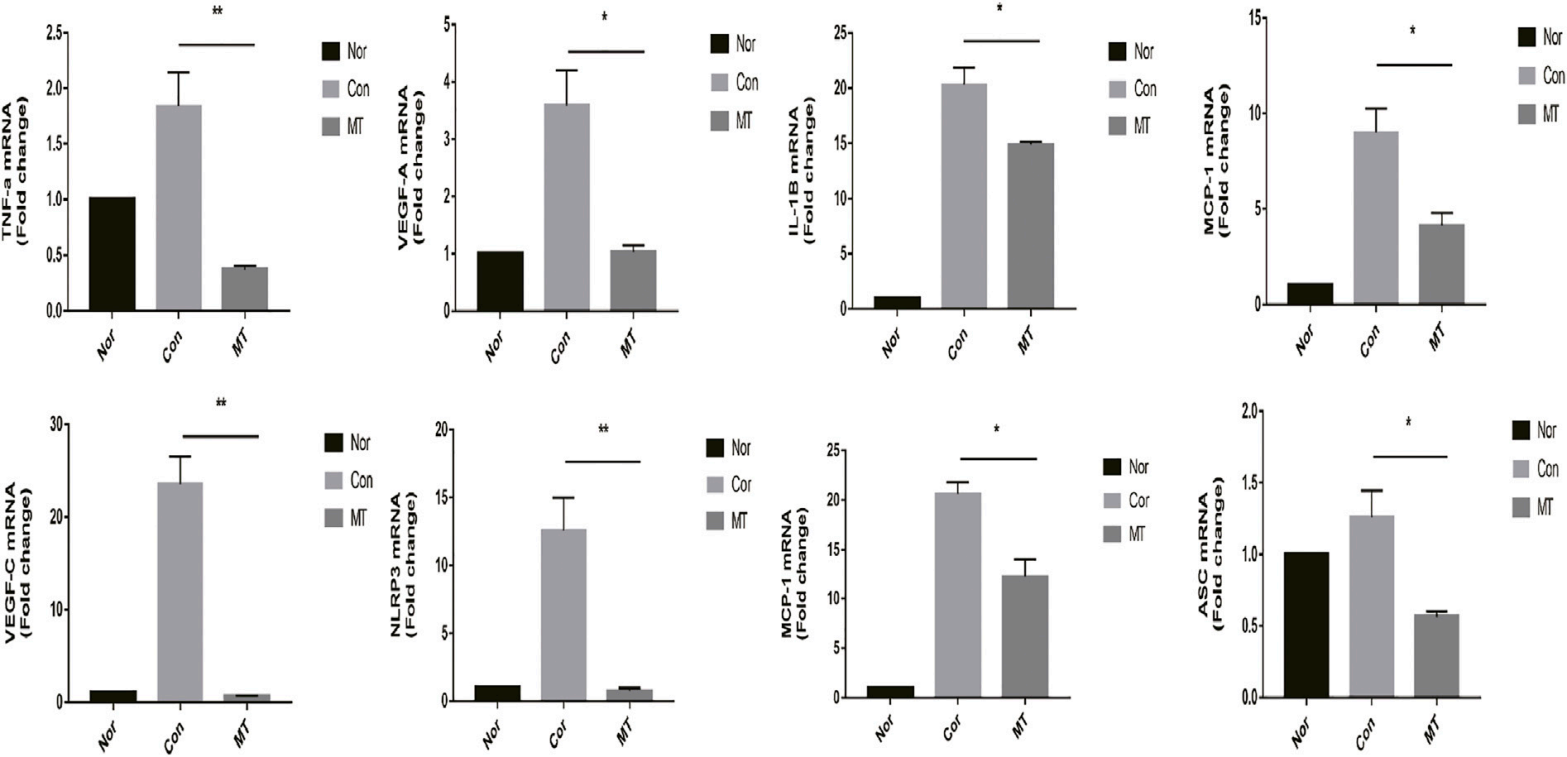
MT inhibits the expression of chemokines and proinflammatory factors in the cornea. On the 9th day after surgery, the mRNA expression levels of relevant cytokines in the grafts were determined by quantitative RT-PCR (*n* = 4 per group). (Nor: Normal group; Con: control group; MT: treated with MT). *p < 0.05, **p < 0.01, ***p < 0.001.

Inflammatory cells, such as macrophages, regulate the formation of new blood vessels by releasing large amounts of chemotactic and pro-inflammatory cytokines (Wu et al., 2012). Melatonin displays anti-inflammatory and antioxidant properties that can influence tissue growth and apoptosis. Studies have shown that MT reduces organ and species dependence and decreases graft rejection. Therefore, we studied the effect of MT on the expression of chemokines and pro-inflammatory factors in transplant rejection, and further explored the mechanism of MT in transplant rejection. Our results showed the expression levels of chemokines, including MIP-1 and MCP-1, pro-inflammatory cytokines (IL-1β and TNF-α), NLRP3, VEGF-A, and ASC, were up-regulated in corneal transplants (Figure 3).

### MT Regulates Treg/Teff Balance in the Body

**FIGURE 4:**
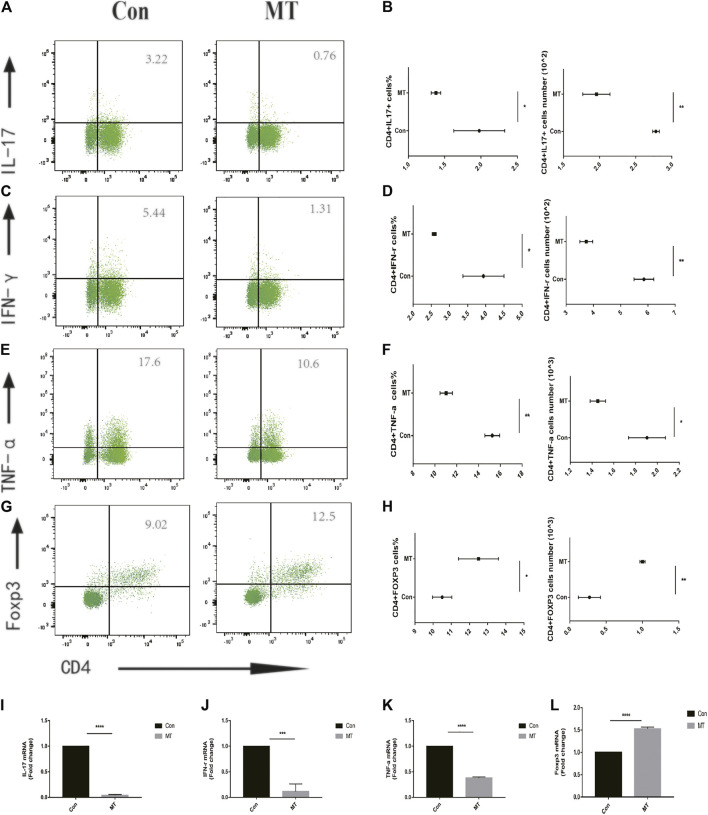
MT regulates Teff/Treg balance in corneal graft model mice. **(A–F)** On the 9th day after operation, the levels of Teff/Treg cells in DLNs were measured by flow cytometry. MT was found to inhibit the proportion of Th1/Th17 cells in DLNs (*n* = 6 per group). **(G–H)**, while treatment increased the proportion of Treg cells in DLNs (*n* = 6 per group). **(I–L)** Real-time quantitative PCR was used to measure the expression level of inflammatory related mRNA. MT inhibited the mRNA expression of IL-17, IFN-γ, TNF-α, and Foxp3 (*n* = 4 per group). (Con: control group; MT: treated with MT). *p < 0.05, **p < 0.01, ***p < 0.001.

Effector T cells, including Th1 and Th17 cells, play a major role in corneal graft rejection. Therefore, we evaluated the effect of MT treatment on Treg/Teff immune cell balance *in vivo*. In animal models of postoperative graft rejection, flow cytometry showed that MT increased the frequency and number of Tregs in DLNs (Figures 4A–G). Meanwhile, MT reduced the frequency and number of Th1/Th17 cells compared with the control group, as shown in (Figures 4B–H).

### MT Inhibits the Expression of Chemokines and Pro-Inflammatory Factors in RAW264.7 Cells *in vitro*

**FIGURE 5:**
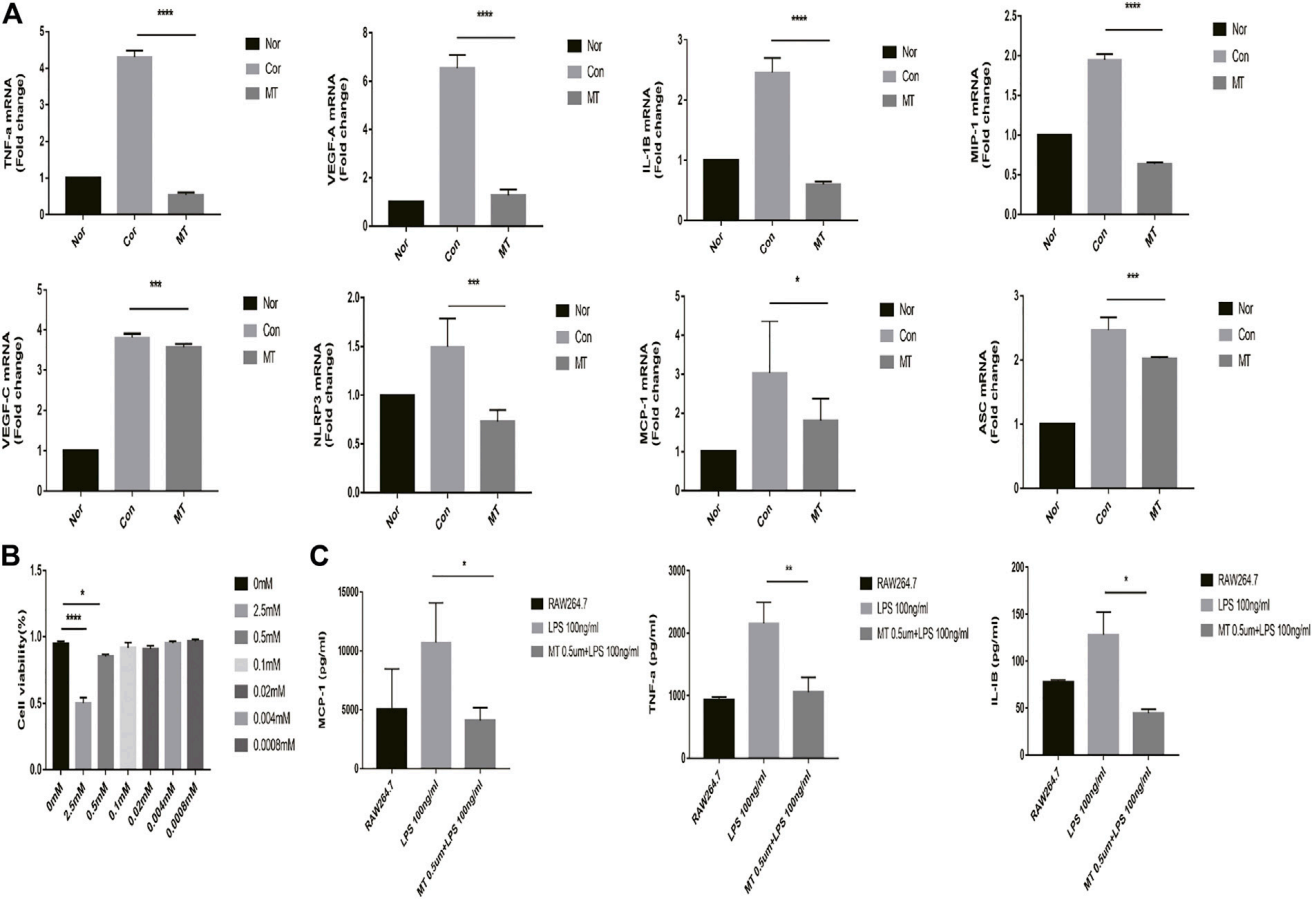
MT inhibits the expression of inflammatory cytokines in RAW264.7 macrophages. **(A)** mRNA expression of inflammatory cytokines was measured by real-time quantitative PCR (*n* = 4 per group). **(B)** The cell viability was determined by CCK8 assay (*n* = 4 per group). **(C)** Protein expression of inflammatory cytokines in culture supernatants was measured by ELISA (*n* = 4 per group). (Nor: Normal group; Con: control group; MT: treated with MT). *p < 0.05, **p < 0.01, ***p < 0.001.

We measured the viability of RAW264.7 cells after MT intervention with the CCK8 assay. The cells were seeded in a 6-well plate at a density of 2 × 10^5^ cells/well and were treated with a gradient concentration of MT for 4 h, and then subsequently treated with 100 ng/ml LPS for 24 h. High concentrations of MT affected the viability of RAW264.7 cells (*p* < 0.05, Figure 5B); However, most macrophages, present at low concentrations, survived.

RAW264.7 cells were incubated with MT for 6 h and subsequently treated with LPS for 4 h; the expression of chemokines and proinflammatory factors was then measured. The qPCR and ELISA results showed that melatonin (500 mM) could inhibit the expression of inflammatory cytokine mRNA and protein (*p* < 0.05, Figures 5A,C).

## Discussion

In order to explore a more effective and safe treatment for transplant rejection, we used MT to treat corneal transplant mice to study its potential therapeutic effects and underlying mechanisms. During these experiments, the mice were intraperitoneally injected with 50 mg/kg MT, as described previously (Chang et al., 2016). Our results are the first to demonstrate that MT promotes corneal allograft survival. This conclusion was based on the following observations. First, MT treatment decreased the number of macrophages *in vivo and in vitro*, and reduced the transcription level of inflammatory cytokines. Second, MT could prolong the survival time of experimental mouse corneal transplant rejection, reduce lymphangiogenesis and corneal edema, and inhibit inflammatory cell infiltration. Finally, MT could regulate the balance of Treg/Teff in the draining lymph nodes of mice.

The inflammation is considered to be a key participant in acute and chronic allograft rejection. In the completely mismatched heart transplant model, compared with the allograft control group, the heart allograft with rejection in the grafts, the expression of ASC increased significantly, and this expression pattern mimicked the expression of IL-1β (Seto et al., 2014). In a retrospective study of 1,271 kidney transplant patients, NLRP3 inflammasomes were significantly positively correlated with the risk of acute rejection (Dessing et al., 2016). These studies indicate that inflammasomes may represent a key target to reduce the incidence of acute rejection. NLRP3 plays a key role as a possible target for transplant rejection intervention and reduces the rate of dysfunction of chronic allografts, and thus provides a potential clinical treatment strategy for corneal transplantation. In this study, MT was found to inhibit NLRP3 inflammasome activation. MT acts as an effective anti-inflammatory agent to treat whole joint replacement by inhibition of NLRP3 (Wu et al., 2021).

The innate immune system is the first barrier to the immune defense (Ploeger et al., 2013). Activated macrophages are inflammatory cells with antigen presentation and costimulatory functions, and the role of the APC, expressed by macrophages in corneal graft immunity, mainly includes interactions with adaptive immune cells such as T cells, triggering a cascade and postcorneal graft rejection (Oh et al., 2013). In addition, recent studies have shown that immune-rejected corneal grafts show a high number of inflammatory macrophages, which are involved in the initial stages of transplantation rejection, and which are scarce in non-rejected grafts (Oh et al., 2013). It has been demonstrated that removal of rat macrophages can completely prevent corneal transplant rejection during a 3-month follow-up period (Veen et al., 1994). A previous study showed that targeting small molecules to inhibit the NLRP3 inflammasome secreted by macrophages can significantly improve the rejection of mouse skin allografts (Amouzegar et al., 2016). In this study, we investigated the possible regulatory effect of MT on the NOD-like receptor protein 3 (NLRP3) inflammasome-mediated release of IL-1β from macrophages. We found that macrophage activity was down-regulated in the MT-administered group. It is generally believed that the autophagy mechanism induced by LPS is closely related to the activation of NLRP3 inflammasomes (Tian et al., 2021). In addition, we showed in the inflammatory macrophage model that different concentrations of MT can have the same inhibitory effect on macrophage activation in cells *in vitro*. Our *in vitro* study showed that MT upregulated IL-1 secretion by blocking the NLRP3 inflammasome activation.

T cells, located in the DLNs of the transplant recipient, are activated by antigen-presenting cells, a mechanism which is characteristic of organ rejection (Wieland and Shipkova, 2015). Immune imbalance between Tregs and Teffs (Tahvildari et al., 2016) has also been shown to aggravate transplant rejection (Zhang et al., 2016). Teffs are a type of pro-inflammatory T cell, predominantly encapsulating Th1/Th17 cells. The increase in the number of Teff cells in corneal transplant patients exacerbates the progression of immune rejection (Yin et al., 2015). Treg cells exhibit protective and anti-inflammatory effects, and transplant rejection in corneal transplant patients has been suggested to be related to the decrease in the frequency and abundance of Tregs (Bian et al., 2021). A recent study found that activation of the NLRP3 inflammasome exacerbates corneal graft rejection through subsequent T cell imbalance (Wei et al., 2020). NLRP3 expression promotes monocyte differentiation, and activated macrophages further secrete MCP-1, recruiting more monocytes and/or macrophages to the site of wound and further activating T cells. In our study, MT inhibited T cell differentiation, promoted Treg polarization, weakened Teff cell polarization, and reduced NLRP3 expression of mouse transplanted corneas. Our results suggest that MT regulates the transition from innate to adaptive immune responses in transplant rejection.

Lymphangiogenesis plays a key role in the immune response, and newly formed lymphatic vessels increase the risk of graft rejection after subsequent corneal transplantation (Jiang et al., 2004). In previous studies, we discovered a parallel development of corneal angiogenesis and lymphangiogenesis after keratoplasty and corneal lymphatic vessels might play a more important role than blood vessels in allograft rejection. On the one hand, corneal lymphangiogenesis enables the exit of antigenic material, antigen-presenting cells (APCs) and so on from the graft to the regional lymph node, and accelerate the rejection, which result in corneal edema. On the other hand, corneal lymphatic vessels are conducive to the reabsorption of tissue fluid and reduce corneal edema. In our experiment, we found that corneal lymphatics decreased and corneal edema also decreased. We concluded that this was because the effect of corneal lymphatics mediated immune rejection was much greater than its function of absorbing tissue fluid.

In the current study, we observed decreased corneal lymphangiogenesis in MT-treated mice, which could have resulted in an increase in allograft survival. Recently, it has been demonstrated that corneal lymphangiogenesis is induced by macrophages infiltrating the cornea. Depletion of macrophages inhibits corneal lymphangiogenesis and corneal graft rejection (Maruyama et al., 2005). Moreover, studies have shown that among tumor-infiltrating immune cells, macrophages promote lymphangiogenesis via NLRP3-dependent IL-1β secretion (Benjamin Weichand et al., 2017). Our work showed that MT inhibits NLRP3-dependent IL-1β secretion by macrophages, which might be partially responsible for the decrease in corneal lymphangiogenesis in MT treatment mice.

In this experiment, BALB/C mice were selected as the donor and C57BL/6 as the recipient. We observed more serious inflammation and anterior adhesion and higher rejection rate of the recipient than in previous experiments. In the study, two of the eight allografts remained non-rejected in the MT group, whereas none of the eight allografts survived in mice treated with physiological saline, which means that the graft rejection rate of this corneal transplantation model is 100%. Relevant studies have shown that the number of spontaneously formed lymphatic vessels and activated macrophages is significantly higher in C57BL/6 cornea than in the BALB/C cornea, both of which are risk factors for rejection (Wei et al., 2020). In our study, we found both corneal lymphangiogenesis and angiogenesis occurred in normal C57BL/6 mice corneas (Figure 1D). Therefore, corneal transplantation performed on C57BL/6 “cornea bed” is regarded as a “high-risk” model of corneal transplantation.

Our research is limited by the following facts: due to the lack of experimental samples, we lack the control group of autologous syngeneic corneal transplantation. In addition, we need to further explore the optimal therapeutic concentration and treatment mode of MT, as well as toxic and side effects. Further studies await to elucidate it.

Based on the above experimental data, we conclude that MT inhibits the activation of NLRP3 inflammasomes to reduce corneal transplant rejection, and that this effect is related to macrophage suppression, T cell regulation, and the reduction in corneal lymphangiogenesis. In summary, MT may be a promising treatment for individuals who undergo corneal transplants.

## Data Availability

The raw data supporting the conclusions of this article will be made available by the authors, without undue reservation.
